# Structural Studies on the Binding Mode of Bisphenols to PPARγ

**DOI:** 10.3390/biom14060640

**Published:** 2024-05-30

**Authors:** Abibe Useini, Inken Kaja Schwerin, Georg Künze, Norbert Sträter

**Affiliations:** 1 Institute of Bioanalytical Chemistry, Centre for Biotechnology and Biomedicine, Leipzig University, Deutscher Platz 5, 04103 Leipzig, Germany; abibe.useini@bbz.uni-leipzig.de; 2 Institute for Drug Discovery, Leipzig University, Brüderstraße 34, 04103 Leipzig, Germany; inken.schwerin@medizin.uni-leipzig.de; 3 Interdisciplinary Center for Bioinformatics, Leipzig University, 04107 Leipzig, Germany; 4 Center for Scalable Data Analytics and Artificial Intelligence (ScaDS.AI), Leipzig University, 04105 Leipzig, Germany

**Keywords:** peroxisome proliferator-activated receptor gamma, bisphenols, obesity, adverse health, structural analysis

## Abstract

Bisphenol A (BPA) and bisphenol B (BPB) are widely used in the production of plastics, and their potential adverse health effects, particularly on endocrine disruption and metabolic health, have raised concern. Peroxisome proliferator-activated receptor gamma (PPARγ) plays a pivotal role in metabolic regulation and adipogenesis, making it a target of interest in understanding the development of obesity and associated health impacts. In this study, we employ X-ray crystallography and molecular dynamics (MD) simulations to study the interaction of PPARγ with BPA and BPB. Crystallographic structures reveal the binding of BPA and BPB to the ligand binding domain of PPARγ, next to C285, where binding of partial agonists as well as antagonists and inverse agonists of PPARγ signaling has been previously observed. However, no interaction of BPA and BPB with Y437 in the activation function 2 site is observed, showing that these ligands cannot stabilize the active conformation of helix 12 directly. Furthermore, free energy analyses of the MD simulations revealed that I341 has a large energetic contribution to the BPA and BPB binding modes characterized in this study.

## 1. Introduction

Obesity is a widespread epidemic impacting both adults and children. It poses a significant risk for the development of physical and mental disorders and is a threat to public health. The number of overweight and obese people has escalated to alarming levels, with 40% of the global adult population being affected. According to the World Health Organization, the incidence of obesity has nearly tripled since 1975. As of 2016, more than 1.9 billion adults were overweight, and over 650 million adults were classified as obese [1]. Emerging research suggests that endocrine disruptors contribute to the rise in obesity rates. Exposure to these chemicals, which are found in many everyday products, can alter metabolic processes and increase the risk of obesity [2,3]. Both in vivo and in vitro investigations have demonstrated the promotion of adipogenesis, lipid accumulation and acceleration of pre-adipocyte differentiation as a result of exposure to these chemicals used as plasticizers [4,5,6,7,8]. They interfere with the body’s hormonal system and with metabolic pathways that play a key role in regulating hormonal activity, lipid homeostasis and adipogenesis. Among other pathways, adipogenesis is regulated by peroxisome proliferator-activated receptor gamma (PPARγ) signaling [9,10].

PPARγ, an intensively studied nuclear receptor (NR), is intricately involved in many essential pathways, including adipocyte differentiation and the regulation of glucose and lipid metabolism [11]. Upon forming heterodimers with retinoic acid receptor (RXR), PPARγ orchestrates the transcriptional regulation of various genes. The signaling activity of PPARγ can be fine-tuned by ligands, both endogenous and synthetic, which interact with the ligand binding domain (LBD) of PPARγ [12,13,14,15,16,17]. Besides the LBD, PPARγ contains a DNA-binding domain and a hinge region. Given its significance in drug design and pharmaceutical treatments, the structure and ligand interactions of PPARγ LBD have been extensively studied. It consists of 13 α-helices and a four-stranded β-sheet. In contrast to other NRs, PPARγ features an additional α-helix, named H2′, positioned between the initial β-strand and H3. Notably, the orientation of H2 in PPARγ differs from other NR structures, thereby facilitating enhanced ligand accessibility. H3, H7 and H10 form a large cavity for ligand binding [13]. The flexible Ω loop between H3 and H2′ may serve as a gate influencing access of ligands to the binding pocket [18].

Effective ligand-dependent gene transcription relies on a highly conserved motif, termed activating function-2 (AF-2). Positioned at the C-terminus of the ligand binding domain, this motif resides within a large Y-shaped ligand binding pocket (LBP), encompassing the AF-2 sub-pocket adjacent to helix 12 and the Ω sub-pocket [13,19]. The binding of ligands to the LBP of PPARγ promotes complex formation with co-activator proteins binding to the AF-2 surface (outside of the LBP). This in turn favors the formation of the heterodimer with RXR, DNA binding and transcription of the target genes [20]. The activation mechanism of PPARγ by endogenous lipids, including fatty acids or their metabolites such as 15-deoxy-delta-12,14-prostaglandin J2 (15d-PGJ2) and synthetic compounds such as thiazolidinediones, has been characterized by X-ray crystallography [12,13,14,15,19,21].

Bisphenols have been extensively utilized in the plastic industry as plasticizers for over half a century. Bisphenol A (BPA) and related bisphenols are mostly used in the production of polycarbonate plastics, epoxy resins and thermal papers [22]. BPA was first synthesized in 1891 and has been used since the 1950s–1960s in plastic fabrication, although its estrogenic properties have been known since the 1930s [23]. BPA became one of the chemicals produced in the highest volume worldwide, reaching a global consumption of 5.6 million metric tons in 2022 [24]. The increasing use of BPA led to a closer investigation and biomonitoring of human exposure. BPA is non-covalently dispersed in plastic polymers and diffuses into food and drinks. Therefore, exposure to BPA is believed to primarily occur through diet. Food warming in plastic containers accelerates BPA leaching. Ingestion is not the only root for BPA uptake. It can occur also through skin penetration and inhalation.

The amount of BPA in urine and serum has been assessed by several studies for different groups of people [25]. Studies report average BPA levels in urine in a range from 0.2–5.7 ng/mL. Based on the 2013–14 National Health and Nutrition Examination Survey (NHANES), urinary BPA is detectable in over 90% of the United States population, with children having significantly higher BPA levels than adults [26]. In the latest release of the BPA re-evaluation by the European Food Safety Authority (EFSA) in 2023, the tolerable daily intake of BPA for humans was lowered from 4 μg to 0.2 ng per kilogram of body weight per day (kg/bw/day). People with average or high exposure exceed this threshold, indicating health concerns [27]. The use of BPA is now limited not just in items designed for infants (EU Commission Regulation 2018/213) [28] but also in other food contact materials and other products, such as thermal paper, where it should not exceed 0.02% by weight [22]. BPA is substituted by other bisphenols like bisphenol B, C, AF, F, S.

With the restriction of BPA, the use of BPB as a substitute is gradually increasing. BPB is a very close structural analog of BPA and is more resistant to biodegradation [29]. This stresses the need to assess its potential endocrine properties and to evaluate if it is safer than BPA. BPB has been detected in human urine, blood and serum [30,31,32], food and the environment [29,33,34,35]. BPB was found at a serum level of up to 5.15 ng/mL mean concentration, which is higher than BPA (concentration 2.91 ± 1.74 ng/mL) [36].

BPB has been investigated for health risks by many studies, and BPB effects on reproductive function have been reviewed [37]. Exposure of zebrafish to BPB induced developmental toxic effects and oxidative damage [36]. Another study showed that BPB exposure adversely affects the uterus morphology in mice [38]. Furthermore, BPB is known to exert higher estrogenic effects than BPA, which is mediated through the G protein-coupled estrogen receptor pathway [39]. In the cited study, the binding affinity of BPB to GPER was 9-fold higher compared to that of BPA.

BPA induces a significant increase in pre-adipocyte proliferation, lipid accumulation and increased expression of PPARγ [8,16,40]. A study on murine pre-adipocytes concluded that BPA and BPS induce adipogenesis through direct activation of PPARγ [41]. In contrast to this, a decrease in adipogenesis of human adipocytes and antagonistic effects of bisphenol A and B on human PPARγ signaling were reported [16]. The presence of BPA did not promote adipogenesis in mesenchymal stromal stem cells (MSCs), in contrast to 3T3-L1 cells, where induced adipogenesis was observed already at 10 nM BPA concentration [42]. An antagonistic effect on rosiglitazone activation of PPARγ was observed at 10 μM of BPA. Activation of PPARγ depends on the degree of halogenation of BPA analogs [43]. Brominated BPA derivatives exhibited increasing potency and agonistic activity on PPARγ signaling with an increasing degree of bromination. For tetrachlorobisphenol A (TCBPA) and tetrabromobisphenol A (TBBPA), half-maximal inhibition (IC_50_) of rosiglitazone binding (at 3 nM) to cells expressing PPARγ was detected at 0.7 μM and 6 μM, respectively [43]. In a surface plasmon resonance assay, an increasing signal was observed in the range of 25 to 400 μM BPA or BPB [16].

Tetrachlorobisphenol A (TCBPA) and tetrabromobisphenol A (TBBPA) bind in very similar binding modes to the LBP of PPARγ [43]. Furthermore, co-crystal structures of ERRγ, which is another member of the group of nuclear receptors, with BPA (PDB 2P7G) and BPB (PDB 6I61) are available, too. ERRγ and PPARγ have similar folds and a sequence similarity of 26% (Table S1). In addition to bisphenols, other plasticizers or their metabolites such as mono(ethylhexyl) phthalate (MEHP) and 1,2-cyclohexanedicarboxylic acid mono-4-methyloctyl ester (MINCH), have been identified as agonists of PPARγ. We previously determined the binding modes of MEHP and MINCH to PPARγ [17].

In this work, we aimed to study the binding modes of BPA and BPB on PPARγ and compare them with those of TCBPA, TCBPB, MEHP, MINCH and other PPARγ ligands (Figure S1). We co-crystallized PPARγ with BPA or BPB and determined the complex structures by X-ray crystallography. Molecular dynamics (MD) simulation was used to study the stability of the binding modes and the ligand interactions with the coordinating protein side chains.

## 2. Materials and Methods

### 2.1. Chemicals 

For PPARγ LBD expression, the gene was obtained by gene synthesis from Invitrogen. Restriction enzymes and buffers used for subcloning were purchased from New England Biolabs (Frankfurt am Main, Germany). The pre-packed columns for protein isolation were ordered from Cytiva (Freiburg, Germany). Chemicals for buffers were from either Roth (Karlsruhe, Germany) or Sigma Aldrich (Taufkirchen, Germany). 4,4′-isopropylidendiphenol (Bisphenol A, 97% purity, Cas-No.: 80057, Product Number: 133027) and 2,2-Bis-(4-hydroxyphenyl)-butan, 4,4′-*sec*-butylidendiphenol (Bisphenol B, Cas-No.: 77407, Product Number: 50877, 98% purity) were purchased from Sigma Aldrich (Taufkirchen, Germany).

### 2.2. Protein Expression, Purification and Characterization

Human PPARγ LBD was prepared as described before [17]. Briefly, PPARγ was expressed for 4 hours, in 3 L *E. coli* culture. After harvesting and cell lysis, PPARγ was isolated via the His_6_-tag, using a 5 mL HiTrap-chelating-Ni^2+^ (Cytiva) column. Thereafter, PPARγ was purified to homogeneity using size exclusion chromatography. The pure protein was concentrated to 10 mg/mL and stored flash-frozen at −80 degrees. Sample purity and quality were assessed via SDS-PAGE and dynamic light scattering.

### 2.3. Crystallization and Structure Determination

For co-crystallization of PPARγ with BPA and BPB, roughly 800 different crystallization conditions were screened in 96-well format plates. These experiments were performed by sitting drop vapor diffusion with a drop volume of 200 nL and a reservoir volume of 100 μL using a Mosquito Xtal3 pipetting robot (SPT Labtech, Melbourn, England). The conditions giving the best crystals were manually reproduced at a microliter scale, in a hanging drop vapor diffusion setup, mixing equal volumes of protein sample and reservoir solution (1 µL each). Prior to crystallization, the protein sample was concentrated at 10 mg/mL in PBS (phosphate-buffered saline) buffer pH 7.4 and mixed with 1 mM peptide PPARγ co-activator 1α (PGC-1α, QEAEEPSLLKKLLLAPANT) and BPA or BPB to a final concentration of 15 mM. Both ligands were prepared as 1 M stock solutions in DMSO. PPARγ × BPA crystals grew at 19 °C in 20% *w*/*v* PEG 3350 and 0.2 M magnesium acetate as crystallization reservoir, whereas PPARγ × BPB crystals grew in a reservoir solution composed of 20% *w*/*v* PEG 3350 and 0.2 M NaCl. Both PPARγ × BPA and PPARγ × BPB crystals appeared after about 5 days.

Single crystals of 0.5–1 mm length were flash-frozen in liquid nitrogen without cryoprotectant. Data were collected at beamline P14 at PETRA III, at the DESY synchrotron (Hamburg, Germany). The diffraction data were indexed, integrated and scaled with XDS (version 10 January 2022) [44] and STARANISO (version 2.3.74) [45] as implemented in ISPyB [46] at DESY. Statistics of data collection and refinement are listed in Table S2. The structure of unliganded PPARγ of PDB id 8BF1 [17] was used as a starting model for rigid-body refinement (REFMAC version 5.8.0425, [47]) of PPARγ × BPA. For the PPARγ × BPB structure, the protein chains were placed by molecular replacement using Phaser (version 2.8.3) [48] as implemented in CCP4i2 (of CCP4 version 8.0) [49] using PDB id 8BF1 as the search model. Both crystals belong to a P2_1_ crystal form that has been observed in several PPARγ crystal structures before and which contains one PPARγ molecule in the asymmetric unit. Helix H12 is in the active (“up”) conformation. The structures were further refined with jelly-body refinement. Coot (version 0.9.8.93) [50] was used for model building, and phenix (version 1.20.1_4487) [51] for the final refinement steps. The stereochemical restraints for BPA and BPB were generated with the Grade Web Server (https://grade.globalphasing.org, accessed on 16 January 2023). Similar to previous PPARγ co-crystal structures with natural ligands such as 15d-PGJ2 or nonanoic acid [21], the electron density of the ligands is weak, most likely due to partial occupancy and flexibility. The ligand occupancy was refined to 0.58 (BPA1), 0.61 (BPA2) and 0.89 (BPB).

### 2.4. Molecular Dynamic Simulations

The procedure used for the MD simulations is summarized in Scheme S1. The PPARγ × BPA co-crystal structure was used as the initial protein model to generate all protein complex models for the MD simulations. Three complex models were generated: (1) a model (denoted as PPARγ_BPA) with the two BPA binding modes observed in the PPARγ × BPA crystal structure, (2) a model (denoted PPARγ_BPA-TCBPA) with BPA bound in the binding mode observed for PPARγ × TCBPA crystal structure (PDB id 3osi) [43] and (3) a model (denoted PPARγ_BPB) of the BPB binding mode as observed in the PPARγ×BPB crystal structure. Starting from the PPARγ × BPA co-crystal structure, alternate side-chain conformations with the lower occupancy were removed, and crystallographic water molecules that had either no contact with other atoms isolated or too short distances to non-hydrogen atoms (d ≤ 2.4 Å) were removed. The structure was further prepared via the structure preparation and protonate3D modules in MOE (version 2022.02) [52] to place polar hydrogen atoms. This yielded the PPARγ_BPA model with two bound BPA molecules (BPA1 in the LBP and BPA2 outside of the LBP). To create the input model for the PPARγ_BPA-TCBPA system, BPA was superimposed onto the TCBPA binding mode via real space refinement in the TCBPA electron density using the program COOT [50]. This complex was then superimposed onto the PPARγ_BPA model based on the Cα coordinates of the protein chains. The BPA-TCBPA ligand was inserted into the model, and clashing water molecules and the two BPA ligands of the PPARγ_BPA model were removed. For modeling PPARγ_BPB, the PPARγ × BPB crystal structure was superimposed onto the PPARγ_BPA model, and the BPB ligand was inserted into the protein model. Again, the two BPA molecules and clashing water molecules were deleted. Thus, for all three simulations, the same protein model was used as the starting structure.

MD simulations of the PPARγ structure bound to BPA1 and BPA2, BPA-TCBPA and BPB were performed using Amber20 [53]. The ff19SB force field was used for the protein [54], and the general Amber force field (GAFF) for the ligand [55]. The assignment of Amber atom types for the BPA and BPB ligands was performed using Antechamber (version 21.0) [56]. RESP atomic charges of the ligands were calculated using Gaussian 16 [57] and Antechamber [56]. The PPARγ–ligand complex structure was surrounded by a cubic TIP3P water box with a thickness of at least 13 Å between any protein or ligand atom and the edge of the box. Crystallographic waters that did not clash with other water molecules or protein atoms were kept in the model. The charge of the system was neutralized by adding Na^+^ or Cl^−^ ions. SHAKE bond length constraints [58] were applied to all bonds involving hydrogen atoms. Nonbonded interactions were evaluated with a 10 Å cutoff, and electrostatic interactions were calculated by the particle-mesh Ewald method [59].

The energy of the MD system was first minimized using a two-step minimization procedure: 20,000-step minimization of water molecules and ions and 20,000-step minimization of the whole system. With protein and ligand atoms restrained to their minimized coordinates, the system was then heated from 0 K to 300 K over 75 ps in the NVT ensemble with a step size of 1 fs. After changing to the NPT ensemble, the system was equilibrated at 300 K with a reference pressure of 1 bar for 10 ns using weak positional restraints (with a force constant of 10 kcal mol^−1^ Å^−2^) applied to protein backbone and ligand heteroatoms. Langevin dynamics with a collision frequency of 1 ps^−1^ and an integration time step size of 2 fs were used in these steps. Positional restraints on protein and ligand atoms were then removed stepwise in a total of 15 ns and the system was equilibrated for another 5 ns without Cartesian restraints. Production MD was conducted for 100 ns using constant pressure periodic boundary conditions and Langevin dynamics. Three independent replicas were carried out for each PPARγ × ligand complex.

The binding free energy (ΔG_binding_) of the BPA and BPB ligands as well as the per-residue contributions to ΔG_binding_ were computed using the MM/GBSA procedure of the MMPBSA.py program (version 14.0) [60]. To this end, molecular conformations were sampled at 50 ps intervals from the last 95 ns of each MD simulation, corresponding to 5700 conformations per system, and used to compute the molecular mechanics energy and solvation free energies. The single trajectory mode was applied; i.e., snapshots of the protein, ligand, and protein–ligand complex were taken from the same trajectory. The ionic strength of water was set to 150 mM.

Analysis of the MD trajectories was carried out using CPPTRAJ (version 6.4.4) [61]. This included calculation of the root-mean-square deviations (RMSDs) and root-mean-square fluctuations (RMSFs) for protein and ligand and enumeration of protein–ligand hydrogen bonds. Trajectories were visualized and molecular graphics generated using ChimeraX (version 1.6.1) [62].

### 2.5. Conservation Analysis

Previously, we analyzed the LBP of PPARγ for the evolutionary conservation of amino acids by comparing vertebrate ortholog sequences [17] using the ConSurf server [63,64]. For analysis of the residues involved in the binding of BPA and BPB, we used the same analysis for all residues within a 5 Å distance from the ligand.

## 3. Results

### 3.1. Binding of BPA to PPARγ

**Figure 1:**
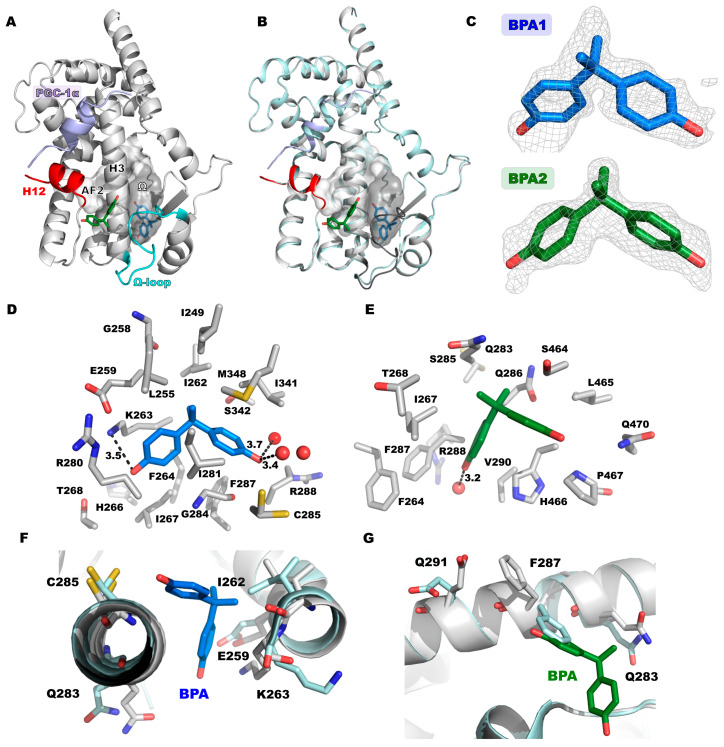
Binding mode of BPA to PPARγ. (**A**) PPARγ × BPA crystal structure with two bound BPA molecules. BPA1 (blue) binds in the Ω sub-pocket (grey, labeled “Ω”), and BPA2 (green) binds outside the LBP. The peptide PGC1α is shown in blue, H12 in red and the Ω loop in cyan. (**B**) Alignment of PPARγ × BPA (grey) with the unliganded PPARγ structure (cyan, PDB: 8BF1). (**C**) Residual difference electron density (polder omit as calculated with phenix) of BPA molecules contoured at 3.2σ_rmsd_ for BPA in Ω sub-pocket (blue) and 3.0σ_rmsd_ for BPA near H12. (**D**) Interactions of BPA in the Ω pocket and near H12 (**E**). (**F**,**G**) Superpositions of the BPA-bound (white) and unliganded (light blue) PPARγ structures at the BPA binding sites in the Ω pocket (**F**) and near helix H12 (**G**). Side chains that are positioned differently in the two structures are shown.

To characterize the binding mode of BPA, the PPARγ LBD was co-crystallized with BPA and PGC-1α co-activator peptide, which contains the motif LXXLL, important for the interaction with the protein. PGC-1α binds near helix H12 and keeps the structure in the active conformation. Crystals of the *P*2_1_ space group with one protein in the asymmetric unit were obtained, which diffracted to 1.4 Å (Figure 1A, Table S2). The overall fold of the structure is almost identical to the apo structure of PPARγ (Figure 1B), with some minor differences in flexible loops. Residual difference electron density indicated the presence of two BPA molecules bound to the LBP (Figure 1A,C). One BPA molecule (BPA1) binds in the Ω sub-pocket of PPARγ. The binding of BPA to this sub-pocket is supported by the Ω loop (residues I262-V277). This loop is flexible and usually not resolved in PPARγ crystal structures. In the PPARγ × BPA structure, the loop could be modeled completely (Figure 1A and Figure S2A,B). Hydrophobic interaction of BPA in the Ω sub-pocket with I262 and K263 stabilizes the loop conformation in addition to crystal packing interactions of H266 and P269. In comparison to the ligand-free structure, I262 has moved out of the ligand binding pocket, ensuring significant space for the bound BPA. Differences are also observed for residues E259 and Q283 (Figure 1F).

BPA in the Ω pocket forms a hydrogen bond with K263 of the Ω loop and with two water molecules. However, the C_δ_, C_ε_ and N_ζ_ atoms of this side chain have weak density indicating flexibility (Figure S2C). The remaining contacts involve essentially hydrophobic interactions (Figure 1D). The second BPA molecule (BPA2) binds at a crystal contact (Figure S3). It binds near helices H3 and H12, interacting with Q286 and F287 of helix H3. The BPA is hydrogen-bonded to a water molecule (Figure 1E). Residues Q283, F287 and E291 are oriented differently than in the apo structure to accommodate the bound BPA (Figure 1G). Both BPA molecules have weaker density compared to the surrounding protein residues (Figure S2C,D). The ligand occupancy was refined to 0.58 (BPA1) and 0.61 (BPA2) with average B factors of 43.1 Å^2^ (BPA1) and 36.3 Å^2^ (BPA2). The average B-value of the protein atoms within a 5 Å distance from BPA1 and BPA2 is 42.7 Å^2^ and 42.3 Å^2^.

### 3.2. Binding Mode of BPB to PPARγ

**Figure 2:**
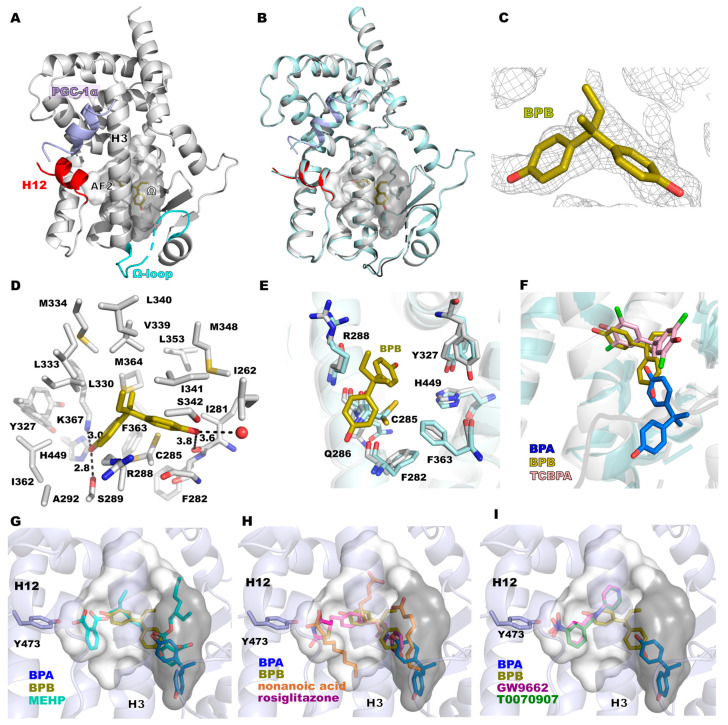
Crystal structure of PPARγ in complex with BPB. (**A**) Overall structure of PPARγ × BPB. Helix 12 is displayed in red, PGC-1α in blue, BPB in yellow and the Ω loop in cyan. (**B**) Superposition of PPARγ × BPB and the apo structure of PPARγ (PDB 8BF1). (**C**) (2Fo-Fc)-type electron density of BPB contoured at 0.71σ_rmsd_ level. (**D**) Interactions of BPB. Distances of polar interactions are specified in Å. (**E**) shows the residues that undergo slight changes upon BPB binding (grey), compared to the apo structure of PPARγ (cyan). (**F**) Superposition of binding modes of BPA, BPB and tetrachloro-bisphenol A (TCBPA, PDB 3OSI). (**G**) Superposition of the PPARγ × BPA, PPARγ × BPB and PPARγ × MEHP (PDB 8BF2) structures. (**H**) Superposition of binding modes of BPA, BPB, nonanoic acid (PDB 4EM9) and rosiglitazone (PDB 4EMA). (**I**) Superposition of binding modes of BPA, BPB, GW9662 (PDB 6MD1) and T0070907 (PDB 61CI). The LBP is displayed as a transparent surface with the AF2 sub-pocket in white and the Ω sub-pocket in grey.

A co-crystal structure of the PPARγ LBD in complex with the co-activator peptide PGC-1 α and BPB was analyzed at a resolution of 1.7 Å. The crystals contain one molecule in the asymmetric unit, and helix H12 is in the active conformation (Figure 2A). A superposition with the unliganded structure of PPARγ shows only differences in loop regions (Figure 2B). The electron density showed the binding of one BPB molecule near C285 (Figure 2A). Weak electron density indicated partial occupancy (refined to 95%) and/or flexibility of the BPB ligand (Figure 2C and Figure S2E). The average B factor of BPB is 38.5 Å^2^, and that of the protein atoms within a 5 Å distance from BPB is 23.3 Å^2^. One of the BPB phenol groups interacts with both helix 3 and helix 7 via S289 and K367, while the other phenol moiety forms weak polar interactions with a water molecule and the backbone of I281. Hydrophobic interactions further support the positioning of BPB in the hydrophobic ligand binding pocket (Figure 2D). The binding of this phenol is probably mostly supported by the π-π-stacking interaction with the peptide bond between G284 and C285. In a comparison to the apo structure of PPARγ (Figure 2E), slight rearrangements of residues surrounding BPB are observed. This includes mostly R288 and C285 (and nearby residues Q286, F282 and F363).

BPB binds similarly to the halogenated BPA derivatives tetrachlorobisphenol A and tetrabromobisphenol A (Figure 2F) [43]. The phenol group that is coordinated to K367 shares the same binding site with the halogenated analogs, whereas the other phenol group is more oriented for an interaction with the side chain of I341.

### 3.3. Molecular Dynamics (MD) Simulations of the BPA and BPB Binding Modes

**Figure 3:**
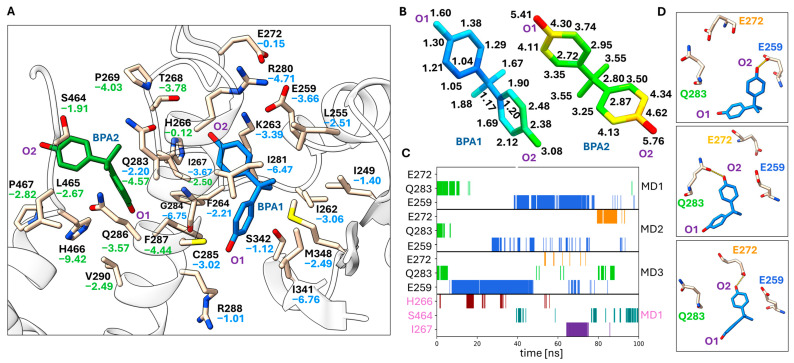
MD analysis of the BPA binding mode. (**A**) Depiction of the BPA binding pocket including the most important residues that take part in ligand binding based on the computed per-residue MM/GBSA energy. Residues are labeled with their respective MM/GBSA energies in kJ/mol (BPA1 blue, BPA2 green). (**B**) RMSF values mapped onto the BPA structures (color coding from blue (low values) over green to red (high values)). The RMSF values in Å are indicated next to the atoms. (**C**) Occurrence of the three most frequent hydrogen bonds of BPA observed over the time course of the three MD replicates for BPA1 and one replicate for BPA2 (pink). (**D**) Representative conformations of BPA1 in the ligand binding pocket. Hydrogen bond interactions are indicated in yellow.

Since the weak density of the BPA and BPB binding modes indicates partial occupancy and alternate conformations and since one of the BPA binding sites (BPA2) is supported by a crystal contact, we decided to study the binding modes of the bisphenol molecules using MD simulations. In addition to the crystallographically observed BPA and BPB binding modes, we also modeled BPA in the binding mode observed for TCBPA [30]. TCBPA binds similarly to BPB near C285 (Figure 1F). The MD simulation of the BPA1 binding mode resulted in a stable binding mode within the 100 ns time period (Figure 3B). The phenol ring hydrogen that is bonded to K263 in the crystal structure (labeled O_2_ in Figure 3) exhibited more flexibility resulting from a switch between three different hydrogen bonding interactions with the side chains of E272, Q283 and E259 (Figure 3C,D). This phenol ring is oriented towards the flexible Ω loop, which might contribute to its flexibility. Hydrophobic interactions with I341, I281 and G284 contribute mostly to the low energy of this binding mode (Figure 3A). The binding mode of BPA2, in contrast, displays much higher RMSF values (Figure 3B). The BPA2 molecule even escaped its binding site in two out of three MD replicates, in one run after ca. 60 ns and in another after ca. 70 ns. The BPA2 molecule only remained at its crystallographically observed binding site in one MD run, making intermittent hydrogen bonds with H266, S464 and I267. For the BPA1 binding mode, the total MM/GBSA energy was −31.0 ± 3.1 kcal/mol, whereas this value amounted to −20.2 ± 3.9 kcal/mol for the BPA2 binding mode.

**Figure 4:**
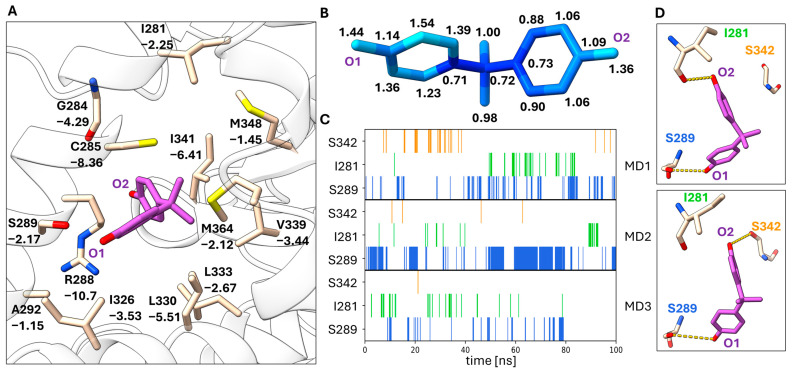
MD analysis of the BPB binding mode. (**A**) Depiction of the BPB binding pocket including the residues that are most important for ligand binding based on the computed per-residue MM/GBSA energies. Residues are labeled with their respective MM/GBSA energies in kJ/mol. (**B**) RMSF values are mapped onto the BPB structure (blue to green to red). The RMSF values in Å are indicated next to the atoms. (**C**) The occurrence of the three most abundant hydrogen bonds of BPB mapped over the time course of three MD replicates. (**D**) Representative conformations of BPB in the ligand binding pocket. Hydrogen bond interactions are indicated in yellow.

MD simulations of the BPB binding mode also resulted in relatively low RMSF values indicating a stable binding mode in the 100 ns time period (Figure 4B). The hydrogen bonding interaction of the O_2_ phenol ring with the side chain of S289 is observed in most frames of the MD simulations, in particular in replicas 1 and 2 (Figure 4C). Other hydrogen bonding contacts are only occasionally observed. Hydrophobic interactions with C285, R288 and I341 contribute most strongly to the stability of this binding mode (Figure 4A). A total MM/GBSA energy of −30.0 ± 2.9 kcal/mol was obtained for the BPB binding mode.

**Figure 5:**
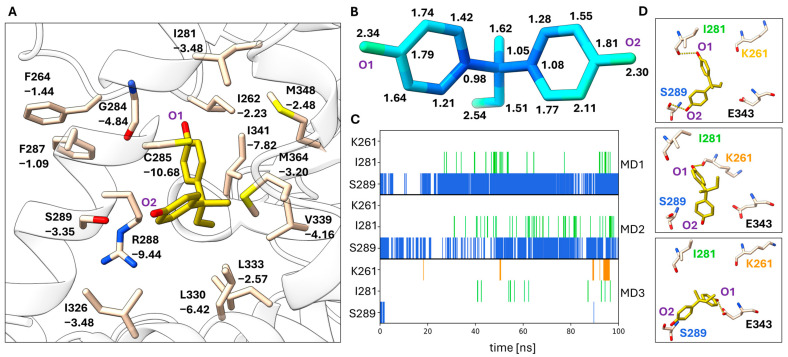
MD analysis of the BPA bound in the TCBPA binding pocket. (**A**) Depiction of BPA in the TCBPA binding pocket including the most important residues for ligand binding based on the computed per-residue MM/GBSA energies. Residues are labeled with their respective MM/GBSA energies in kJ/mol. (**B**) RMSF values are mapped onto the BPA structure (from blue over green to red). The RMSF values in Å are indicated next to the atoms. (**C**) The occurrence of the three most abundant hydrogen bonds of BPA mapped over the time course of three MD replicates. (**D**) Representative conformations of BPA in the TCBPA binding pocket. Hydrogen bond interactions are indicated in yellow.

The binding mode of BPA observed in the PPARγ × BPA crystal structure is stabilized by interactions with the Ω loop, a flexible loop that is fixed in one conformation by crystal packing interactions in this crystal structure. Since these crystals were obtained by co-crystallization of PPARγ with BPA, it might be that ligand binding stabilized the observed conformation of the Ω loop and induced the formation of the obtained crystals. Nevertheless, the BPA binding mode is in turn also indirectly stabilized by crystal packing interactions and it might be that other low-energy binding modes exist in the LBP. For the BPA derivative TCBPA, a binding mode near C285 has been previously observed [30]. Therefore, we modeled BPA in a binding mode resembling that of TCBPA and studied its stability and interactions via MD simulations (Figure 5).

An analysis of the RMSF values shows that this binding mode is the least flexible in the MD simulations (Figure 5B). The TCBPA binding mode is similar to the BPB binding mode (Figure 2F) in particular concerning the phenol group that interacts by hydrogen bonding with S289. This hydrogen bonding interaction is also the most frequently observed hydrogen bonding interaction in the MD simulations of BPA in the TCBPA binding mode (Figure 5C,D). As for BPB, the interactions with C285, R288 and I341 contribute most strongly to this binding mode. A total MM/GBSA energy of −27.9 + −2.3 kcal/mol was obtained for the BPA-TCBPA binding mode.

### 3.4. Conservation of the BPA and BPB Binding Sites in Other Species

Exposure to plasticizers is not only a concern for human health, the leaking of these compounds into the environment might also affect animal wildlife. We therefore studied the conservation of the BPA and BPB binding sites in different species (Table S3) [17]. We checked residues that are within 5 Å distance from each ligand. Following the overall evolutionary distance, these residues are highly conserved in the investigated vertebrates, but differences occur in fish (Figure S4).

## 4. Discussion

Crystal structure analysis of the binding modes of BPA and BPB on PPARγ showed that these ligands bind to the LBP, but fail to interact with Y473 of helix 12. PPARγ ligands which make polar interactions with Y473 can stabilize helix 12 in an active conformation, increasing the affinity for interaction of co-activator proteins binding to the AF2 surface. This is considered a key interaction that determines the activity of full agonists such as fatty acids as endogenous ligands or synthetic agonists such as rosiglitazone (Figure 2H) [13,65,66]. The plasticizer metabolites MINCH and MEHP also interact with Y473 (Figure 2G), and their agonistic activity on PPARγ signaling probably depends on this interaction [17]. Binding of BPA was observed at two sites: (1) in the Ω pocket and (2) outside the LBP (Figure 1). The latter binding mode is supported by direct crystal contacts (Figure S3), and MD simulations indicated a low stability of BPA bound at this site in the absence of crystal contacts (Figure 3B). The magnitude of the calculated total MM/GBSA energy is −20.2 ± 3.9 kcal/mol, significantly smaller in magnitude than that of the other binding modes. These findings indicate that the BPA2 binding mode is not physiologically relevant.

The BPA binding mode in the LBP is supported by the flexible Ω loop, which is stabilized in the PPARγ × BPA crystal structure by crystal packing contacts and interaction with the ligand. In the MD simulations, this binding mode was quite stable, in particular the interaction with I341 and G284, whereas the other phenol group that interacts with the Ω loop was more flexible. We also showed that BPA modeled in the binding mode observed for TCBPA [43] has high stability in the MD simulations, as indicated by its low RMSF values. While the binding energies of the BPA1 and BPA-TCBPA binding modes are overall similar, the former is slightly preferred energetically. This is in agreement with the observation of only the BPA1 binding mode in the PPARγ × BPA crystal structure. The two binding modes are too closely spaced to be occupied simultaneously. It was previously shown that the halogenated BPA derivatives TCBPA and TBBPA activate PPARγ at micromolar concentrations, whereas no significant effect was observed for BPA up to 10 μM concentration in this assay [43]. BPB bound to PPARγ in a similar binding mode to that of TCBPA (Figure 2F), and the MD simulations indicated this binding mode has good stability (Figure 1B).

Although the binding modes and interactions of agonists, antagonists and inverse agonists with PPARγ have been characterized in many studies, it is difficult to predict the influence of the BPA and BPB binding modes observed in this study on PPARγ transactivation activity. This is due to the fact that the conformational changes involved in PPARγ activation of full and partial agonists as well as antagonists or inverse agonists are not well understood. It was previously shown that partial agonists, which fail to stabilize the active helix 12 conformation because of a lack of Y473 interaction, instead stabilize the β-sheet region and helix 3, which was indicated by the kinetics of amide H/D exchange [65]. I341, S342 and M348 are part of the β-sheet region of PPARγ, and the partial agonism of MRL-24 was attributed to these interactions [65]. Interaction with I341 has a large contribution to all three BPA and BPB binding modes, as shown by our per-residue MM/GBSA energy analysis (Figure 3A, Figure 4A and Figure 5A). These interactions and the similarity of the BPB binding mode to that of TCBPA and TBBPA suggest that BPA and BPB may exert partial agonistic activity through their binding modes. On the other hand, it has also been demonstrated that small structural changes in ligand structure such as a methine-to-nitrogen substitution can change an antagonist (GW9662) into an inverse agonist (T0070907) [67]. These compounds covalently attach to C285 and bind near the BPA or BPB binding sites (Figure 2I). Differential interactions of these compounds with R288 have been implicated in the observed differences in PPARγ activity. Interactions with C285 and R288 also contribute strongly to the binding modes of BPB (Figure 4A) and of BPA in the TCBPA site (Figure 5A) in the MD simulations. Differential stabilization of PPARγ conformational states for interaction with co-repressors and co-activators is the basis for the differences between GW9662 and T0070907 in PPARγ transactivation activity [67]. In the present work, we could characterize the interactions of BPA and BPB with the active state of PPARγ stabilized by co-crystallization with a co-activator peptide. This was important in experimental work to obtain well-diffracting crystals. Elucidation of the binding modes of bisphenols paves the way for a deeper understanding of how these compounds influence PPARγ signaling. Sensitive binding assays combined with biophysical studies in solution may clarify these open questions.

## Data Availability

The coordinates and structure factors of the crystal structure analysis have been submitted to the protein data bank with the identifiers 9F7W (PPARγ × BPA) and 9F7X (PPARγ × BPB).

## Supplementary Materials

The following supporting information can be downloaded at https://www.mdpi.com/article/10.3390/biom14060640/s1: Table S1: Alignment of human PPARγ and human ERRγ; Table S2: Diffraction data and refinement statistics; Table S3: Sequence IDs used for the alignment analyses shown in Figure S4; Scheme S1: Overview of the methodology used for the MD simulations. Figure S1: Schematic representation of BPA, BPB, TCBPA, MEHP, MINCH, endogenous (nonanoic acid) and synthetic (rosiglitazone, GW9662, T007090) PPARγ ligands; Figure S2: Electron density of the Ω loop and the ligand binding sites; Figure S3: BPA2 binds at a crystal contact in the PPARγ×BPA structure; Figure S4: Amino acid sequence alignment of selected species for mammalian orthologs (*human*, *dog*, *pig*, *mouse*, *rat*, and *whale*), for birds (*chick*), for fish (*zebrafish* and *kryma*), and for reptiles (*lizard*).
