# Supplementary material for: Structural Studies on the Binding Mode of Bisphenols to PPARγ
Source: Biomolecules. 2024 May 30;14(6):640. doi: 10.3390/biom14060640 (PMC11202036; doi:10.3390/biom14060640)
Supplement: Supplementary file 1 [file biomolecules-14-00640-s001.zip › biomolecules-3024156-supplementary.pdf]

Supplementary Information

Structural studies on the binding mode of bisphenols to PPARγ

Abibe Useini, Inken Kaja Schwerin, Georg Künze and Norbert Sträter

Table S1. Alignment of human PPARγ and human ERRγ

PPARγ HUMAN	sp|P37231-2|PPARG_HUMAN Isoform 1 of Peroxisome proliferator-activated receptor gamma OS=Homo sapiens OX=9606 GN=PPARG Query ID: lcl|Query_7251043 Length: 272	
		Sequence identity to hPPARγ (%)
ERRγ HUMAN	sp|P62508|ERR3_HUMAN Estrogen-related receptor gamma OS=Homo sapiens OX=9606 GN=ESRRG PE=1 SV=1 Sequence ID: Query_7251045 Length: 237 Range 1: 48 to 232	26

```
hPPARg 80   CQFRSVEAVQEITEYAKSIPGFVNLDLNDQVTLLKYGVHEI-----IYTMLA---SLMNK  131
ERRg   48   .DLADR.L.V-.IGW..H....ST.S.A..MS..QSAWM..LILGVV.RS.SFEDE.VYA  106

hPPARg 132  DGVLISEGQGFMTREFLKSLRKPFGDFMEPKFEFAVKFNALELDDSDLAIFIAVIILSGD  191
ERRg   107  .DYIMD.D.SKLAG---------LL.LNNAILQLVK.YKSMK.EKEEFVTLK.IALANS.  157

hPPARg 192  RPGLLNVKPIEDIQDNLLQALELQLKLNHPESSQLFAKLLQKMTDLRQIVTEHVQLLQVI  251
ERRg   158  SMHIED.EAVQKL..V.HE..QDYEAGQ.M.DPRRAG.M.MTLPL...TS.KA..HFYN.  217

hPPARg 252  KKTETDMSLHPLLQEI  267
ERRg   218  -.L.GKVPM.K.FL.M  232
```

Table S2. Diffraction data and refinement statistics

**Compound**	**PPARγ×BPA**	**PPARγ×BPB**
PDB entry ID	9F7W	9F7X
Final buffer before cryocooling	20% w/v PEG 3350, 0.2 M Mg-acetate, 137 mM NaCl, 8.1 mM Na_2HPO_4, 1.5 mM KH_2PO_4, 2.7 mM KCl, 1 mM peptide, 15 mM BPA, 1.5 % DMSO	20% w/v PEG 3350, 0.337 M NaCl, 8.1 mM Na_2HPO_4, 1.5 mM KH_2PO_4, 2.7 mM KCl, 1 mM peptide, 15 mM BPB, 1.5 % DMSO
Data collection		
Wavelength (Å)	0.9762	0.9755
Resolution (Å)	41.88-1.25 (1.37-1.25)	42.23-1.63 (1.80-1.63)
Resolution aniso (Å)	1.470, 1.253, 1.331	3.503, 1.749, 1.545
Space group	$P2_1$	$P2_1$
Unit cell dimensions (Å;°)	43.80, 54.19, 66.47; 90.000, 107.035 90.000	40.36, 54.62, 66.72; 90.000, 93.598 90.000
Unique reflections	60529 (3027)	14591 (731)
Multiplicity	6.9 (7.1)	6.8 (6.5)
Completeness (%)* spherical/ellipsoidal	74.1 (16.2) / 91.5 (51.3)	40.3 (8.1) / 88.2 (57.6)
Mean I/s(I)	12.8 (1.5)	10.3 (1.4)
R-meas	0.066 (1.318)	0.103 (1.281)
R-merge	0.061 (1.221)	0.095 (1.178)
R-pim	0.025 (0.491)	0.039 (0.497)
$CC_{1/2}$	0.999 (0.615)	0.999 (0.437)
Wilson B (Å^2)	16.96	17.89
Refinement		
Resolution (Å)	41.88-1.25 (1.27-1.25)	42.23-1.63 (1.65-1.63)
R-work	0.1622 (0.2613)	0.2493 (0.4080)
R-free	0.1889 (0.4057)	0.2899 (0.2864)
Number of non-hydrogen atoms, B-value (Å^2)		
Protein	2307, 33.14	2223, 29.11
Heterogen	34, 39.69	18, 35.62
Solvent	339, 37.43	119, 20.91
Ligand occupancy	0.58 (BPA1), 0.61 (BPA2)	0.89
Rmsd bonds (Å), angles (°)	0.008, 0.905	0.004, 0.531
Ramachandran favored, allowed, outliers (%)	99.27, 0.73, 0.00	96.67, 3.33, 0.00
Rotamer outliers (%)	0.38	1.20
MolProbity clashscore	1.05	5.48

*Anisotropic truncation has been used. The first line refers to the spherical and the second line to the ellipsoidal completeness.

Table S3. Sequence IDs used for the alignment analyses shown in Figure S4.

<table>
<tr><td>PPARγ_HUMAN</td><td>sp|P37231-2|PPARG_HUMAN Isoform 1 of Peroxisome proliferator-activated receptor gamma OS=Homo sapiens OX=9606 GN=PPARG Query ID: lcl|Query_1789 Length: 477</td><td></td></tr>
<tr><td colspan="2"></td><td>Sequence identity to hPPARγ (%)</td></tr>
<tr><td>PPARγ_DOG</td><td>>sp|Q4U3Q4|PPARG_CANLF Peroxisome proliferator-activated receptor gamma OS=Canis lupus familiaris OX=9615 GN=PPARG PE=2 SV=1</td><td>99.16</td></tr>
<tr><td>PPARγ_MOUSE</td><td>sp|P37238|PPARG_MOUSE Peroxisome proliferator-activated receptor gamma OS=Mus musculus OX=10090 GN=Pparg PE=1 SV=3</td><td>97.90</td></tr>
<tr><td>PPARγ_PIG</td><td>sp|O62807|PPARG_PIG Peroxisome proliferator-activated receptor gamma OS=Sus scrofa OX=9823 GN=PPARG PE=2 SV=1</td><td>98.32</td></tr>
<tr><td>PPARγ_RAT</td><td>sp|O88275|PPARG_RAT Peroxisome proliferator-activated receptor gamma OS=Rattus norvegicus OX=10116 GN=Pparg PE=1 SV=2</td><td>97.90</td></tr>
<tr><td>PPARγ_WHALE</td><td>tr|A0A2Y9M8F2|A0A2Y9M8F2_DELLE Peroxisome proliferator-activated receptor gamma OS=Delphinapterus leucas OX=9749 GN=LOC111168952 PE=3 SV=1</td><td>98.74</td></tr>
<tr><td>PPARγ_CHICK</td><td>tr|A0A0S1NES2|A0A0S1NES2_CHICK Peroxisome proliferator-activated receptor gamma OS=Gallus gallus OX=9031 GN=PPARgamma PE=2 SV=1</td><td>92.63</td></tr>
<tr><td>PPARγ_LIZARD</td><td>>tr|A0A670J836|A0A670J836_PODMU Peroxisome proliferator-activated receptor gamma OS=Podarcis muralis OX=64176 GN=PPARG PE=3 SV=1</td><td>90.99</td></tr>
<tr><td>PPARγ_ ZEBRAFISH</td><td>>tr|A6XMH6|A6XMH6_DANRE Peroxisome proliferator-activated receptor gamma OS=Danio rerio OX=7955 GN=pparg PE=2 SV=1</td><td>63.94</td></tr>
<tr><td>PPARγ_KRYMA</td><td>>tr|A0A3Q3ACA3|A0A3Q3ACA3_KRYMA Peroxisome proliferator-activated receptor gamma OS=Kryptolebias marmoratus OX=37003 GN=PPARG PE=3 SV=1</td><td>66.39</td></tr>
</table>

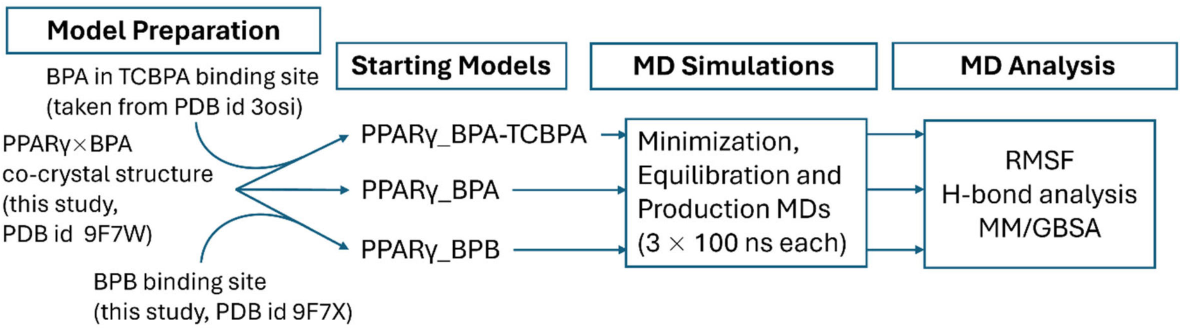

Scheme S1: Overview of the methodology used for the MD simulations.

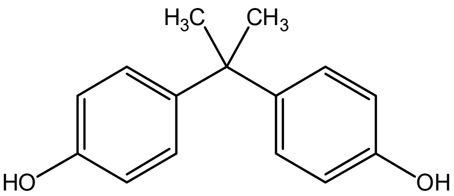

BPA

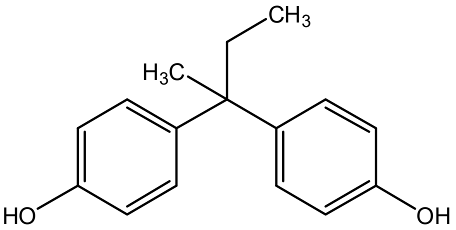

BPB

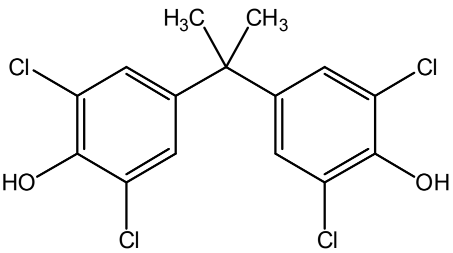

TCBPA

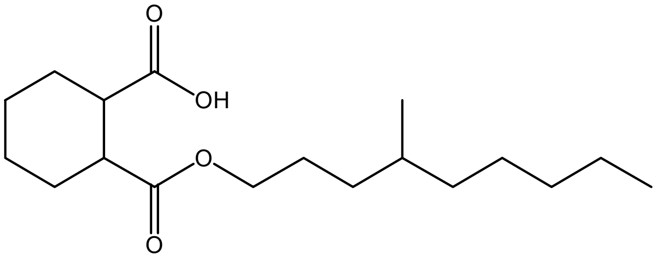

MINCH

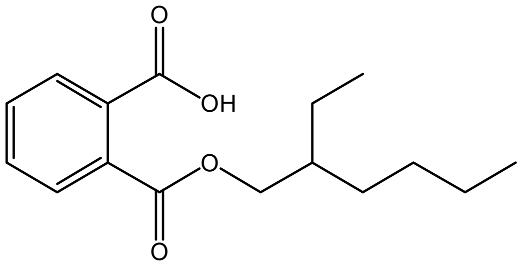

MEHP

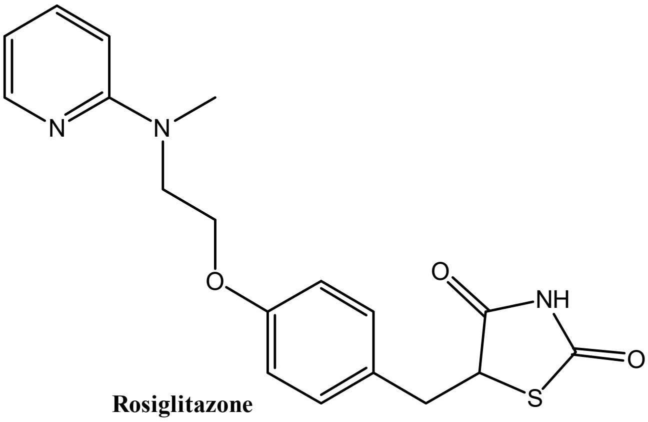

Rosiglitazone

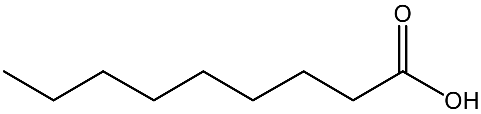

Nonanoic acid

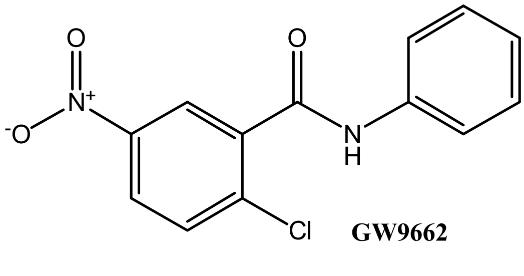

GW9662

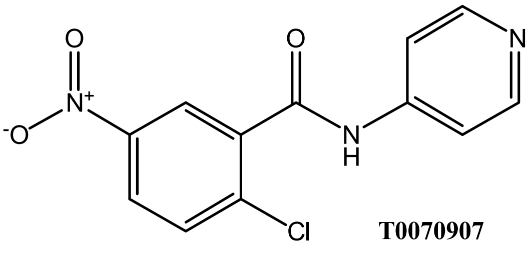

T0070907

Figure S1. Schematic representation of BPA, BPB, TCBPA, MEHP, MINCH, endogenous (nonanoic acid) and synthetic (rosiglitazone, GW9662, T007090) PPARγ ligands.

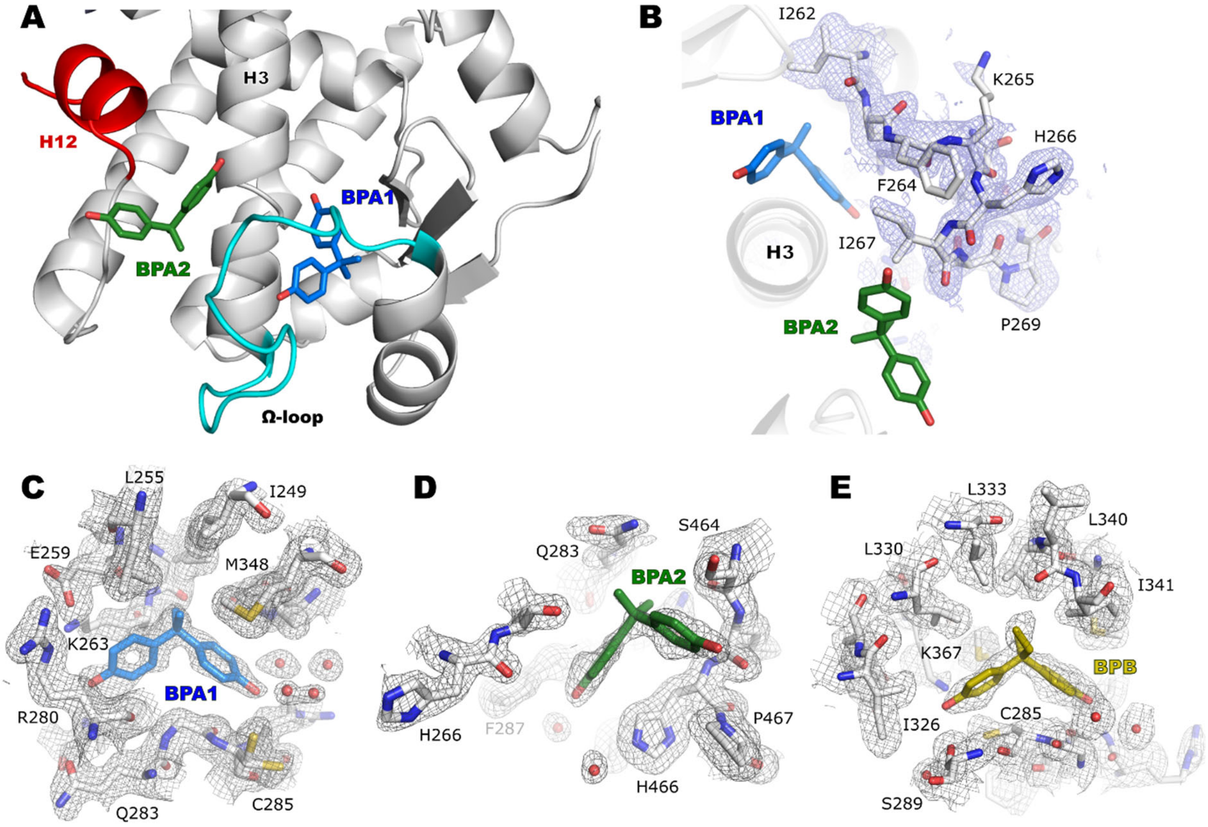

Figure S2. Electron density of the Ω loop and the ligand binding sites. (A) Zoom of the BPA binding sites and the Ω loop. (B) (2Fo-Fc)-type electron density of the Ω-loop at a contour level of 0.5 σ_{rms}. (C-E) (2Fo-Fc)-type electron density maps of the BPA1, BPA2 and BPB binding sites, respectively. The contour level of these maps is 0.7 σ_{rms}.

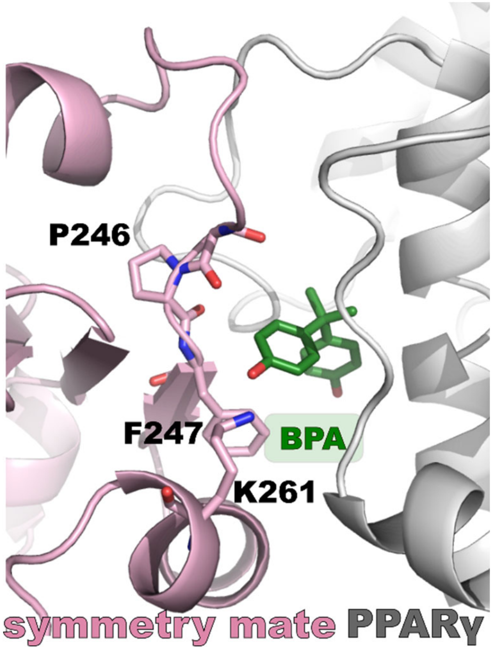

Figure S3. **BPA2 binds at a crystal contact in the PPARγ×BPA structure.** The protein molecule in the asymmetric unit is shown in grey and a symmetry related molecule in pink.

```
    HUMAN  1    MTMVDTE-MPFWPTNFGISSVDLSVMEDHSHSFDIKPFTTVDFSSISTPHYEDIP-FTRT  58
      DOG  30    ......-..................D............................-.S.A  86
      PIG  28   V......-..................D............................-.P.A  85
    MOUSE  29   I......-.......................................A.......-...A  86
      RAT  29   I......-..................D....................A.......-...A  86
    WHALE  1      .....-..................D....................A.......-.P.A  56
    CHICK  1      .....-.....V.....P....A.D..M.................S.......-LG.A  56
   LIZARD  78    ......-.....M.....P..F.G.DE.................L.SQ....L.PL...  135
ZEBRAFISH  17     ....Q-TFG..VG..L.ALE.EEL..DT..L.....S.L.Y....GID..NN.--.QN  71
    KRYMA  1      ....QQLLA..VG.SL.T...PELD.S..CL.M.HLL.S.YA...S-----------S  47

    HUMAN  59   DP------VVADYKY-------------------------DLKLQEYQSAIKVEPASPPY  87
      DOG  87   ..------M......-------------------------....................  115
      PIG  86   ..------M......-------------------------.....D.........V....  114
    MOUSE  87   ..------M......-------------------------....................  115
      RAT  87   ..------M......-------------------------....................  115
    WHALE  57   ..------M......-------------------------....................  85
    CHICK  57   .Q------TSI....-------------------------.I...DC.....M..P....  85
   LIZARD  136  .Q------MTV....-------------------------...F..C.NE..I..P....  164
ZEBRAFISH  72   ..TPHMMDLTHM.S.RTQENYRTHEPIYRPEHSSYSPEENTYRA.QI.NS..L..E...Q  131
    KRYMA  48   SI------PSSF.RT-------------------------EQDTHSKPNS..L..E...Q  76

    HUMAN  88   YSEKTQLYNKPHEEPSNSLMAIECRVCGDKASGFHYGVHACEGCKGFFRRTIRLKLIYDR  147
      DOG  116  ............................................................  175
      PIG  115  ............................................................  174
    MOUSE  116  .........R..................................................  175
      RAT  116  .........R..................................................  175
    WHALE  86   ............................................................  145
    CHICK  86   F...V.........S.....V.......................................  145
   LIZARD  165  F...V.M.......T.............................................  224
ZEBRAFISH  132  FA.NSVSFS.TP.D..S.SLN...................................V..H  191
    KRYMA  77   ..D-SPVFS.FPDDT.ATTLN...................................V..H  135

    HUMAN  148  CDLNCRIHKKSRNKCQYCRFQKCLAVGMSHNAIRFGRMPQAEKEKLLAEISSDIDQLNPE  207
      DOG  176  ............................................................  235
      PIG  175  ............................................................  234
    MOUSE  176  ............................................................  235
      RAT  176  ............................................................  235
    WHALE  146  ............................................................  205
    CHICK  146  ............................................................  205
   LIZARD  225  ............................................................  284
ZEBRAFISH  192  ...H....................M........................F...VNHMH..  251
    KRYMA  136  .E.H....................N........................F...MEH.H..  195

    HUMAN  208  SADLRALAKHLYDSYIKSFPLTKAKARAILTGKTTDKSPFVIYDMNSLMMGEDKIKFKHI  267
      DOG  236  ............................................................  295
      PIG  235  ............................................................  294
    MOUSE  236  ............................................................  295
      RAT  236  ............................................................  295
    WHALE  206  ............................................................  265
    CHICK  206  ................................................R....Q..C..A  265
   LIZARD  285  .........L.......................A..............K....Q..C..L  344
ZEBRAFISH  252  ........R...E..L.Y............S...S.NA....H..K..VE..QM.NCRYM  311
    KRYMA  196  A.......R...EA.L.Y............S...GE.V....H..K...E..QF.NCRQ.  255
```

Figure continued on next page

Figure S4. Amino acid sequence alignment of selected species for mammalian orthologs (human, dog, pig, mouse, rat, and whale), for birds (chick), for fish (zebrafish and kryma), and for reptiles (lizard). The alignment was done using BLAST program (Altschul et al. 1997). Residues involved in binding sites of BPA and BPB are highlighted in red.

```
     HUMAN  268  TPLQEQSK-------EVAIRIFQGCQFRSVEAVQEITEYAKSIPGFVNLDLNDQVTLLKY  320
       DOG  296  ........-------.............................................  348
       PIG  295  ........-------..........................N..................  347
     MOUSE  296  ........-------..........................N....I.............  348
       RAT  296  ........-------..........................N....I.............  348
     WHALE  266  .....P..-------.............................................  318
     CHICK  266  S.....N.-------........R..............F..N..................  318
    LIZARD  345  ..M...N.-------........R..............F..N..................  397
 ZEBRAFISH  312  -..H.HRRSDLGIMH..EL.F.HSY.S..A..IS.V..F.......I.............  370
     KRYMA  256  PS.DH.QQ-------VSTL.F.HS..S..A...R.V..F.......ID............  308

     HUMAN  321  GVHEIIYTMLASLMNKDGVLISEGQGFMTREFLKSLRKPFGDFMEPKFEFAVKFNALELD  380
       DOG  349  ............................................................  408
       PIG  348  ............................................................  407
     MOUSE  349  ..................................N.........................  408
       RAT  349  ............................................................  408
     WHALE  319  ............................................................  378
     CHICK  319  ........L.............D.................C...................  378
    LIZARD  398  ........L...M.........N.................C...................  457
 ZEBRAFISH  371  ..I.VMII.ISP......T...Y..I..............CEM.......SI...M....  430
     KRYMA  309  ..I.VLII.MSP......T...Y..I..............CQM.......S....T....  368

     HUMAN  381  DSDLAIFIAVIILSGDRPGLLNVKPIEDIQDNLLQALELQLKLNHPESSQLFAKLLQKMT  440
       DOG  409  ............................................................  468
       PIG  408  ............................................................  467
     MOUSE  409  ......................................................V.....  468
       RAT  409  ......................................................V.....  468
     WHALE  379  ............................................................  438
     CHICK  379  ............................................................  438
    LIZARD  458  ....................I.......................................  517
 ZEBRAFISH  431  .C.M.L.L.............D......L.ETV.HS......I...D.L.....V.....  490
     KRYMA  369  ...M.L.L...................QL.ETV.HS..........D.L...........  428

     HUMAN  441  DLRQIVTEHVQLLQVIKKTETDMSLHPLLQEIYKDLY  477
       DOG  469  .....................................  505
       PIG  468  .....................................  504
     MOUSE  469  .............H.......................  505
       RAT  469  .............H.......................  505
     WHALE  439  .....................................  475
     CHICK  439  ..............I......................  475
    LIZARD  518  ..............I......................  554
 ZEBRAFISH  491  ....L..D....I.MM.E..A.W.........MR...  527
     KRYMA  429  .......D..H.IELL....V..F........M....  465
```

Figure S4 (continued)
